# PD-1/PD-L1 Interaction Maintains Allogeneic Immune Tolerance Induced by Administration of Ultraviolet B-Irradiated Immature Dendritic Cells

**DOI:** 10.1155/2016/2419621

**Published:** 2016-07-31

**Authors:** Lanfang Zhang, Chang-Qing Xia

**Affiliations:** ^1^Department of Hematology, Xuanwu Hospital, Capital Medical University, Beijing 100053, China; ^2^Department of Chemotherapy, Weihai Municipal Hospital, Weihai 264200, China; ^3^Department of Pathology, Immunology and Laboratory Medicine, University of Florida, Gainesville, FL 32610, USA

## Abstract

Our previous study demonstrated that transfusion of ultraviolet B-irradiated immature dendritic cells (UVB-iDCs) induced alloantigen-specific tolerance between two different strains of mice. Programmed death-1 (PD-1) and programmed death ligand-1 (PD-L1) have been suggested to play an important role in maintaining immune tolerance. In the present study, we seek to address whether PD-1/PD-L1 plays a role in the maintenance of UVB-iDC-induced tolerance. We first observe that the UVB-iDC-induced alloantigen-specific tolerance can be maintained for over 6 weeks. Supporting this, at 6 weeks after tolerance induction completion, alloantigen-specific tolerance is still able to be transferred to syngeneic naïve mice through adoptive transfer of CD4+ T cells. Furthermore, skin transplantation study shows that the survival of allogeneic grafts is prolonged in those tolerant recipients. Further studies show that PD-1/PD-L1 interaction is essential for maintaining the induced tolerance as blockade of PD-1/PD-L1 by anti-PD-L1 antibodies largely breaks the tolerance at both cellular and humoral immunological levels. Importantly, we show that PD-1/PD-L1 interaction in tolerant mice is also essential for controlling alloantigen-responding T cells, which have never experienced alloantigens. The above findings suggest that PD-1/PD-L1 plays a crucial role in maintaining immune tolerance induced by UVB-iDCs, as well as in actively controlling effector T cells specific to alloantigens.

## 1. Introduction

The major obstacle of allogeneic transplantation is the allograft rejection due to mismatched major histocompatibility complex (MHC) antigens [1, 2]. Induction of immune tolerance across MHC barrier is an ideal approach for preventing allograft rejection. It has been demonstrated that steady-state cell apoptosis during self-renewal plays an important role in maintaining immune tolerance to self-antigens [3, 4]. In line with this, we have successfully induced immune tolerance to alloantigens between two different mouse strains through injection of ultraviolet B- (UVB-) irradiated immature dendritic cells (UVB-iDCs) and infusion of iDCs without UVB irradiation mounts potent immune response to alloantigens [5, 6]. Using this approach, we were able to significantly prevent graft-versus-host disease in a mouse model of allogeneic hematopoietic stem cell transplantation [5]. However, how this UVB-iDC-induced tolerance is maintained remains to be determined.

The interaction of programmed death-1 (PD-1) and its ligand (PD-L1) has been proposed to be involved in the modulation of both central and peripheral tolerance [7]. Studies showed that PD-1/PD-L1 interaction was required for both induction and maintenance of T cell tolerance [8–10]. In an alloantigen tolerance induction model, it was shown that PD-1/PD-L1 plays an important role in maintaining long-term allogeneic tolerance induced by infusion of ethylene carbodiimide-fixed allogeneic splenocytes [11]. In our previous study, we demonstrated a significantly prolonged survival in the recipients receiving bone marrow and spleen cells from donor mice tolerant to alloantigens induced by infusion of UVB-iDCs in an allogeneic hematopoietic stem cell transplantation mouse model [5], suggesting that UVB-iDC-induced immune tolerance to allogeneic MHC antigens could be long lasting. In this study, we firstly addressed whether UVB-iDCs treatment-induced alloantigen tolerance could be maintained after induction. Secondly, we addressed whether PD-1/PD-L1 played a role in maintaining this tolerance. The results are reported herein.

## 2. Materials and Method

### 2.1. Mice

8–10-week-old Balb/c (H-2d) and C3H (H-2k) were purchased from Charles River Animal facility (Beijing, China) and housed in the Animal Care facility at Xuanwu Hospital, Capital Medical University, Beijing. All mice were used following the Chinese governmental and Capital Medical University guidelines for animal welfare. This study was approved by the Capital Medical University Animal Ethics Committee. All mice used in this study were euthanized in a CO_2_ chamber with a CO_2_ meter connected to it to control CO_2_ flow as 1.5 L/min.

### 2.2. Dendritic Cell Culture and Preparation

Balb/c bone marrow derived immature dendritic cells (BM-iDCs) were cultured and irradiated by ultraviolet B (UVB) (1200 mJ/cm^2^) following the protocol we reported previously [5, 6]. After being irradiated, iDCs would initiate the process of apoptosis because of the DNA crosslinking induced by UVB irradiation. In our observation, 60–70% of UVB-irradiated iDCs underwent apoptosis 8 h after irradiation and almost all cells died 24 h after irradiation. Therefore, to avoid infusion of apoptotic cells at different stages of apoptosis, the irradiated iDCs were either injected immediately after irradiation or put on ice to stop biological activities of the cells and injected within 2 h.

### 2.3. Alloantigen Tolerance Induction and Adoptive CD4+ T Cells Transfer

As previously reported [5, 6], C3H male mice received intravenous injection of UVB-irradiated Balb/c iDCs (2 × 10^5^ cells/mouse) or PBS once a week for 4 weeks and then plasma anti-donor antibodies were measured by flow cytometry (FCM, Canto, BD) following the protocol described previously [6] to confirm that the tolerance was successfully induced. Six weeks later, we challenged the mice with intravenous injection of 2 × 10^5^/mouse Balb/c spleen cells once a week for two weeks. Thereafter, we measured the anti-donor antibody levels using the method we reported previously [5, 6] with mild modifications. In brief, the anti-donor antibody assay was a flow cytometric analysis of plasma levels of antibodies against donor cells. Donor spleen cells (1 × 10^6^) in 60 *μ*L of PBS were incubated with 40 *μ*L of plasma collected from mice in different experimental groups as indicated and anti-CD4-PercP antibodies at room temperature for 30 min. Then, the cells were washed twice with PBS and resuspended in 100 *μ*L PBS and incubated with secondary antibody, anti-mouse IgG-FITC at room temperature for 30 min. The data were analyzed by flow cytometry. Samples with anti-mouse IgG-FITC staining positive were considered positive for anti-donor antibodies. Using CD4 T cell staining to represent spleen cells was for the purpose of eliminating the false positivity caused by Fc binding of mouse plasma IgG in antigen-presenting cells, mainly B cells.

For CD4+ T cell transfer studies, CD4+ T cells from C3H mice from two different groups used for the above experiments were prepared by negative selection with CD4+ T cell isolation kit (EasySep kit) according to the protocol from the manufacturer (StemCell Biotech). The purity of CD4+ T cells was around 95%. 5 × 10^6^ purified CD4+ T cells were adoptively transferred to naïve C3H mice via tail vein injection. After the injection, all recipient C3H mice were challenged with two weekly transfusions of 2 × 10^5^ Balb/c spleen cells. One week after the second challenge, plasma samples were prepared and assayed for antibodies against Balb/c WBCs. In the above experiments, the mice were monitored twice a week until the endpoint of the experiments and then euthanized as described in Section 2.1.

### 2.4. *In Vivo* Alloantigen-Specific Immune Response Assay and Anti-PD-L1 Antibody Treatment

Tolerant C3H mice received Balb/c spleen cells (1.5 × 10^7^/mouse) prelabeled with carboxyfluorescein diacetate succinimidyl ester (CFSE, from Invitrogen). In the meantime, 100 *μ*g of purified anti-mouse PD-L1 mAb (Biolegend, Clone 10F.9G2) or control IgG (Rat IgG, Sigma) was injected intraperitoneally (i.p.) into these mice, respectively. Nontolerant naïve C3H mice receiving injection of CFSE-labeled Balb/c spleen cells served as an additional control group. Twenty-four hours later, peripheral blood mononuclear cells (PBMNCs) of all recipient mice were prepared and stained by anti-CD4-PercP antibody (BD BioScience, clone RM4-5). CFSE+CD4+ T cells were measured by flow cytometry. The percentage of CFSE+CD4+ T cells in total CD4+ T cells served as a readout to demonstrate the acceptance of donor cells.

For examining the maintenance of humoral immune tolerance, the tolerant recipient C3H mice 6 weeks after tolerance induction received intravenous injection of Balb/c spleen cells (2 × 10^5^/mouse) along with injection of anti-mouse PD-L1 mAb (100 *μ*g) or control IgG (100 *μ*g), once a week for two weeks. Thereafter, plasma samples were prepared and assayed for antibodies against Balb/c WBCs as described above.

In assessing how PD-1/PD-L1 interaction in tolerant C3H mice affected alloantigen-responding T cells from naïve C3H mice, Balb/c spleen cells (1 × 10^7^/mouse) (as alloantigens) and CFSE-labeled naïve C3H spleen cells (1 × 10^7^/mouse) were injected intravenously (i.v.) into tolerant mice with anti-mouse PD-L1 mAb (100 *μ*g/mouse) or control IgG (100 *μ*g/mouse) at day 1 and day 3. Naïve C3H mice receiving injection of both types of cells served as an additional control. On day 4, the recipient mice were sacrificed and inguinal lymph node cells were prepared and stained with anti-CD4 and anti-CD8 fluorescent antibodies, and CFSE-labeled CD4+ and CD8+ T cell proliferation was examined by flow cytometry and analyzed using FCS express software (De Novo software, Vancouver, Canada).

### 2.5. Mouse Skin Transplantation

Recipient C3H mice were prepared with four weekly infusions of UVB-iDCs from Balb/c mice or PBS as described above. To assess the maintenance of tolerance, we performed skin transplantation 6 weeks after finishing tolerance induction. Syngeneic skin transplantation in Balb/c mice was also performed to ensure the success of our surgical procedure. The surgical procedure is as follows: donor skin grafts were from the ears of Balb/c mice. The donor ears were surgically removed and placed in PBS on ice and then split and the dorsal flap was retained for transplant. C3H allogeneic and Balb/c syngeneic recipient mice were anesthetized and placed in lateral position. The transplant area was wetted with 75% alcohol; then the hairs were shaved and the operation area was cleaned with alcohol swab. In the area of skin preparation, 1 cm diameter circular flap was surgically removed and placed on the preprepared graft bed. Donor skin graft was carefully trimmed to fit the graft bed. The recipient mice were wrapped by a sterile bandage around the body to completely cover the surgical area and placed in a clean cage. All recipient animals were monitored on a daily basis. At day 6 after transplant, the bandage was removed and the graft was examined for the success of surgery and graft survival. The recipients were continued to be monitored daily for signs of rejection. When a graft showed signs of scabbing or contraction, skin graft rejection was considered. All mice in these experiments were euthanized using CO_2_ as described in Section 2.1 at the endpoint of the experiment.

## 3. Result

### 3.1. Infusion of UVB-iDCs to Allogeneic Recipients Induces Long-Lasting Alloantigen-Specific Tolerance, Which Is Transferable through Adoptive Transfer of CD4+ T Cells

**Figure 1:**
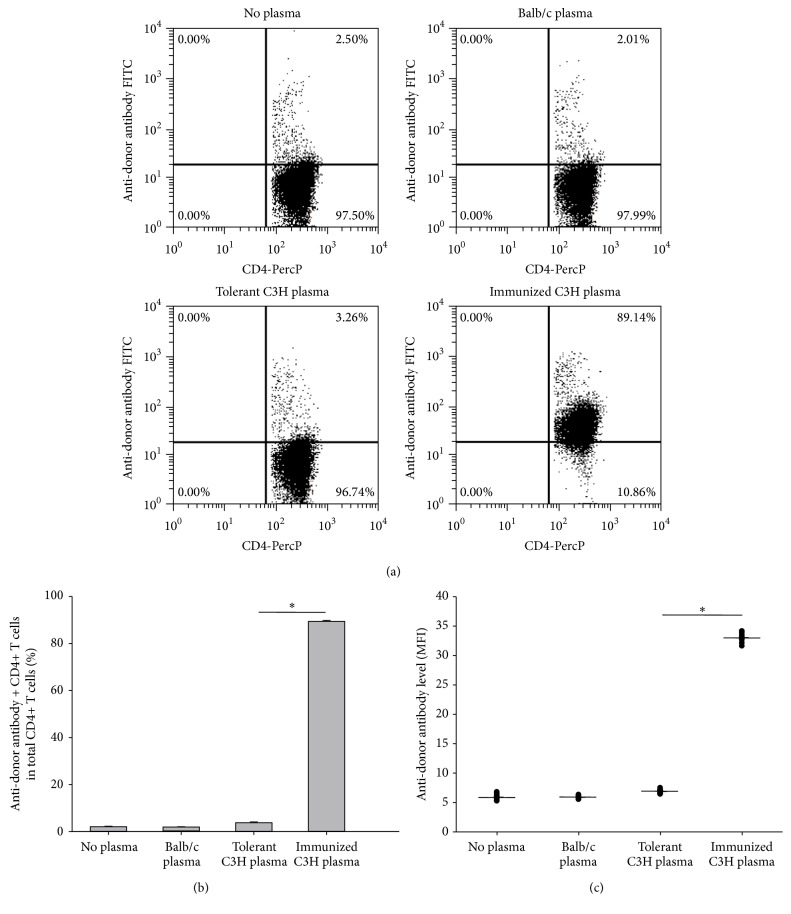
Alloantigen-specific tolerance induced by infusion of UVB-iDCs can be maintained for long term. (a) Recipient C3H mice (H2k) were treated with 4 weekly intravenous injections of donor Balb/c (H2d) UVB-iDCs or PBS (10 mice/group). Six weeks later, all mice were challenged by infusion of Balb/c spleen cells (2 × 10^5^/mouse) once a week for two weeks. One week after the second challenge, plasma levels of anti-donor antibodies were examined. Naïve Balb/c mouse plasma samples were utilized for testing the background binding. Balb/c spleen cells with plasma added were blank controls. ((b) and (c)) The summary of anti-donor antibodies levels in both percentage and mean fluorescent intensity (MFI) is depicted, respectively. Data represent mean ± standard error. ^*∗*^ *P* < 0.05. The *P* values were calculated using Student's *t*-test.

**Figure 2:**
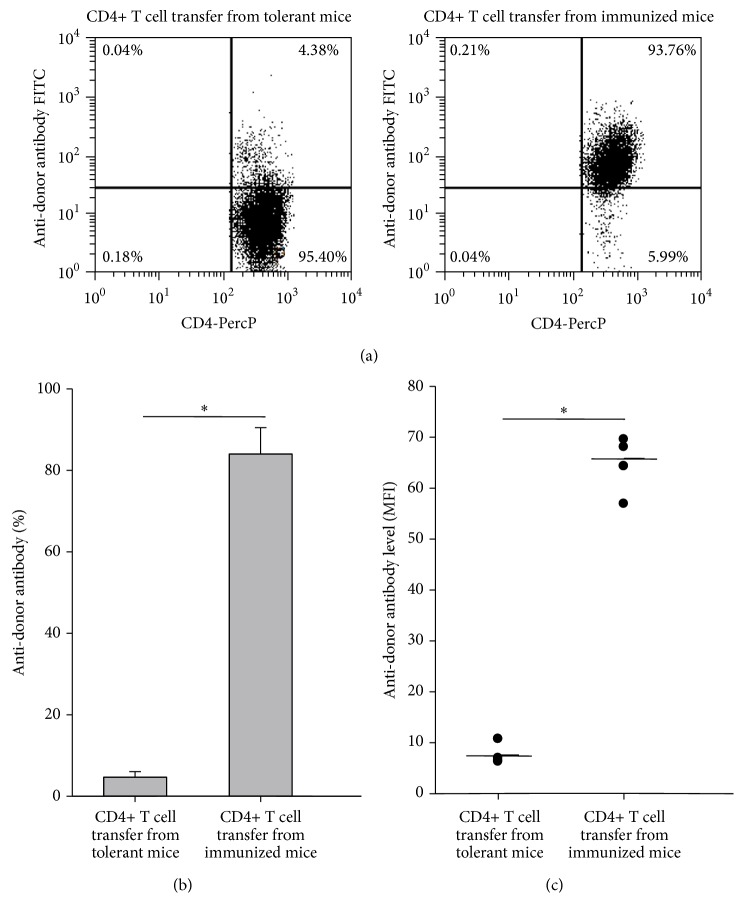
Alloantigen immune tolerance is transferable by adoptive transfer of CD4+ T cells from long-lasting tolerant mice. Splenic CD4+ T cells from C3H mice used for the experiments in Figure 1 were prepared by negative selection using Easysep kits from StemCell Biotechnology Company. Given that all mice had been challenged by injection of Balb/c spleen cells, the PBS-treated C3H mice were all immunized as shown in Figure 1, and the two groups were designated as tolerant group and immunized group, respectively. CD4+ T cells were adoptively transferred into naïve C3H mice (5 × 10^6^ CD4+ T cells/mouse) (5 mice/group). One day after CD4+ T cell transfer, all mice were challenged with two weekly transfusions of 2 × 10^5^ Balb/c spleen cells. One week after the second antigen challenge, the development of antibodies against donor Balb/c WBCs was examined as described in Section 2. (a) A representative of each group was shown. ((b) and (c)) The summary of anti-donor antibody levels in percentage and MFI of each group was depicted, respectively. Data represent mean ± standard error. ^*∗*^ *P* < 0.05. The *P* values were calculated using Student's *t*-test.

Our previous study showed that four weekly transfusions of UVB-iDCs to allogeneic recipient mice induced tolerance to alloantigens [6]. In our allogeneic hematopoietic stem cell transplantation model, it appeared that this tolerance was maintained in the recipient mice 3 months after transplantation [5]. In these experiments, we sought to determine whether this tolerance could be maintained for a period of time after tolerance induction. We followed the same protocol reported previously [6], but, instead of performing assessment 1 week after the last treatment, we evaluated tolerance 6 weeks after finishing tolerance induction through alloantigen challenge by intravenous injection of allogeneic Balb/c spleen cells. We found that C3H mice tolerized by Balb/c UVB-iDC treatment remained tolerant to alloantigen challenge (Figures 1(a), 1(b), and 1(c)), whereas nontolerant C3H mice pretreated by injection of PBS developed high-level anti-Balb/c antibodies in response to Balb/c alloantigen challenge (Figures 1(a), 1(b), and 1(c)). Similar to the results reported previously that CD4+ T cells from the tolerant mice could transfer tolerance [5], we found that this long-lasting immune tolerance could also be transferred to syngeneic naïve C3H mice through adoptive transfer of CD4+ T cells from the tolerant mice, demonstrating that CD4+ T cells from tolerant mice markedly suppressed alloantigen-induced anti-donor antibody development compared to CD4+ T cells transferred from immunized mice (Figures 2(a), 2(b), and 2(c)). The above findings suggest that UVB-iDCs infusion-induced alloantigen immune tolerance can be maintained for long term, and CD4+ T cells play an important role for the maintenance of alloantigen tolerance.

### 3.2. Skin Graft Survival in Tolerant Mice Is Significantly Prolonged

**Figure 3:**
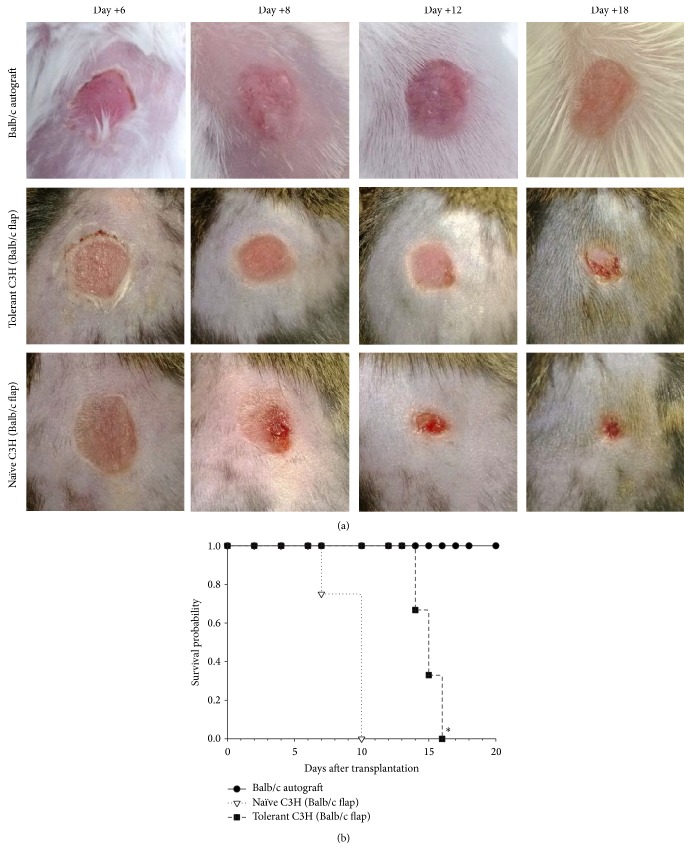
Tolerance induced by injection of UVB-iDCs prolongs the skin allograft survival. Six weeks after the last treatment for tolerance induction as described elsewhere, the tolerant and naïve C3H mice were transplanted with Balb/c skin grafts as described in Section 2. The graft survival was monitored daily for 30 days. (a) The condition of a representative transplanted flap from each group is exhibited. (b) The graft survival of each group was shown. Six mice were included in each group. The survival data were analyzed by Log Rank test. ^*∗*^ *P* < 0.05 (tolerant group versus naïve group).

To further address the strength and maintenance of alloantigen-specific tolerance induced by UVB-iDC treatment, we performed allogeneic skin transplantation in tolerant and PBS-treated C3H mice 6 weeks after tolerance induction. The results shown in Figures 3(a) and 3(b) demonstrated that skin graft survival was significantly prolonged in tolerant mice compared to nontolerant mice. The skin grafts were eventually rejected from the tolerant recipients (Figure 3(b)), suggesting that the tolerance was not sufficient in sustaining allogeneic skin graft survival in this most difficult organ transplantation model. As expected, syngeneic skin grafts were all survived (Figure 3), indicating no technical difficulty concurring in our skin transplantation experiment.

### 3.3. PD-1/PD-L1 Blockade Breaks the Allogeneic Immune Tolerance Induced by Infusion of UVB-iDCs

**Figure 4:**
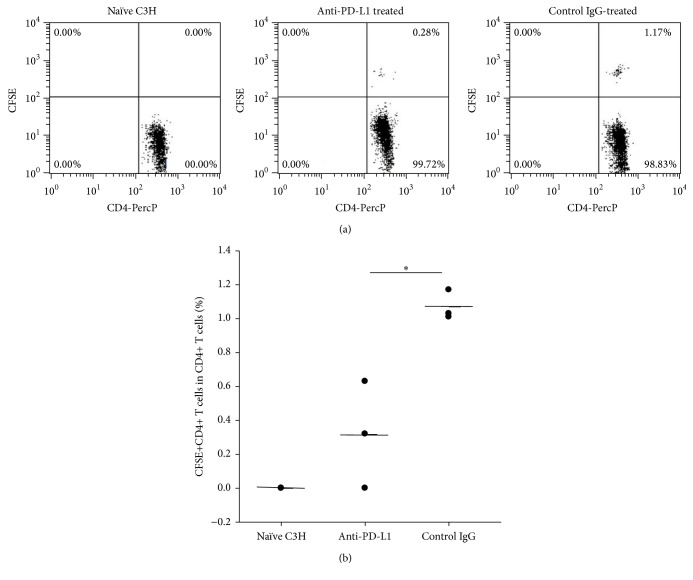
Anti-mouse PD-L1 antibody treatment enhances the rejection of intravenously injected Balb/c spleen cells in C3H mice with long-lasting tolerance. The tolerant C3H mice received purified anti-mouse PD-L1 mAb (100 *μ*g/mouse) or control IgG (100 *μ*g/mouse) along with administration of CFSE-labeled Balb/c spleen cells (1.5 × 10^7^/mouse). Nontolerant C3H mice received the same numbers of CFSE-labeled Balb/c spleen cells as control. Three mice were included in each group. 24 hours after injection, peripheral blood samples were collected from all mice and stained with anti-CD4-PercP antibody. The CFSE+CD4+ cells to total CD4+ T cells were examined by flow cytometry. (a) Flow cytometric data are exhibited from a representative mouse of each group. (b) The data summary of all mice in each group is depicted. Data represent mean ± standard error. ^*∗*^ *P* < 0.05. The *P* values were calculated using one-way ANOVA.

**Figure 5:**
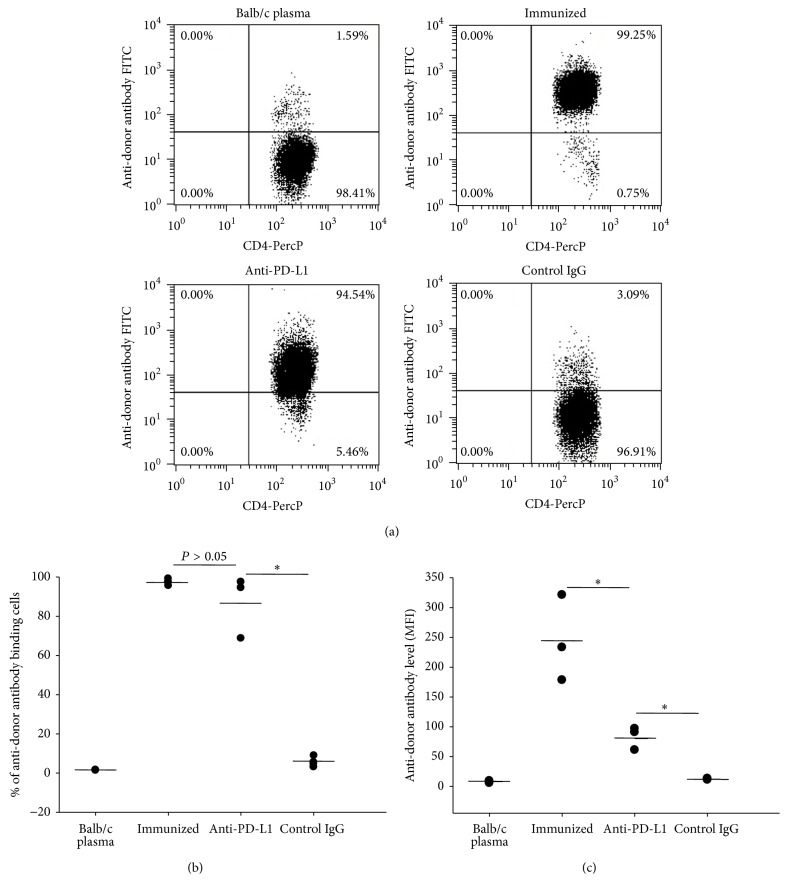
Anti-mouse PD-L1 antibody treatment partially recovers the development of antibodies against alloantigens in C3H mice with long-lasting tolerance to Balb/c alloantigens. Tolerant C3H mice 6 weeks after tolerance induction were given alloantigen challenge by injection of Balb/c spleen cells (2 × 10^5^/mouse) along with anti-mouse PD-L1 mAb (100 *μ*g/mouse) or control IgG (100 *μ*g/mouse) once a week for two weeks. The following week, plasma samples were prepared from all mice, and the levels of anti-donor (Balb/c) antibodies were examined as described elsewhere. The plasma samples from the Balb/c and naïve C3H mice immunized by Balb/c spleen cells served as controls. We only gated CD4+ T cells to analyze anti-donor antibody levels to eliminate false positivity caused by B cell IgG Fc binding. (a) A representative mouse of each group was exhibited. (b) The summary of 3 mice in each group was depicted. Data represent mean ± standard error. ^*∗*^ *P* < 0.05. *P* values were calculated using one-way ANOVA.

To determine whether PD-1/PD-L1 pathway plays a role in UVB-iDC-induced allogeneic tolerance, we employed anti-mouse PD-L1 antibodies to block the PD-1 signaling in the mice with long-lasting tolerance during alloantigen challenge. We intravenously injected fluorescent dye, CFSE-labeled Balb/c spleen cells into C3H mice tolerant to Balb/c alloantigens, with treatment of anti-PD-L1 antibody, or control IgG. Nontolerant naïve C3H mice receiving injection of CFSE-labeled Balb/c spleen cells served as an additional control. In nontolerant control mice, all CFSE-labeled cells were rejected within 24 h after injection (Figures 4(a) and 4(b)). Fair numbers of CFSE-labeled cells remained in the tolerant recipient mice receiving control IgG treatment, whereas these numbers were significantly reduced upon PD-1/PD-L1 blockade by the treatment of anti-PD-L1 antibodies with a complete rejection in one of three mice (Figures 4(a) and 4(b)). Further, we assessed how PD-1/PD-L1 blockade affected anti-alloantigen antibody development upon alloantigen challenge. We showed that tolerant mice receiving control IgG treatment failed to develop any levels of anti-alloantigen antibodies upon alloantigen challenge (Figures 5(a) and 5(b)). However, tolerant mice receiving anti-PD-L1 treatment developed relatively high levels of anti-alloantigen antibodies, which nonetheless were still lower than nontolerant mice challenged with alloantigens based on mean fluorescent intensity (MFI) (Figure 5(c)). This result is consistent with the result shown above that there were still certain numbers of CFSE-labeled cells remaining in some of tolerant mice receiving anti-PD-L1 treatment (Figures 4(a) and 4(b)). These findings suggest that PD-1/PD-L1 plays an important role in maintaining alloantigen tolerance in the tolerant mice.

### 3.4. PD-1/PD-L1 Interaction Is Required to Prevent Naïve Alloantigen-Responding T Cells from Being Activated in the Tolerant Mice

**Figure 6:**
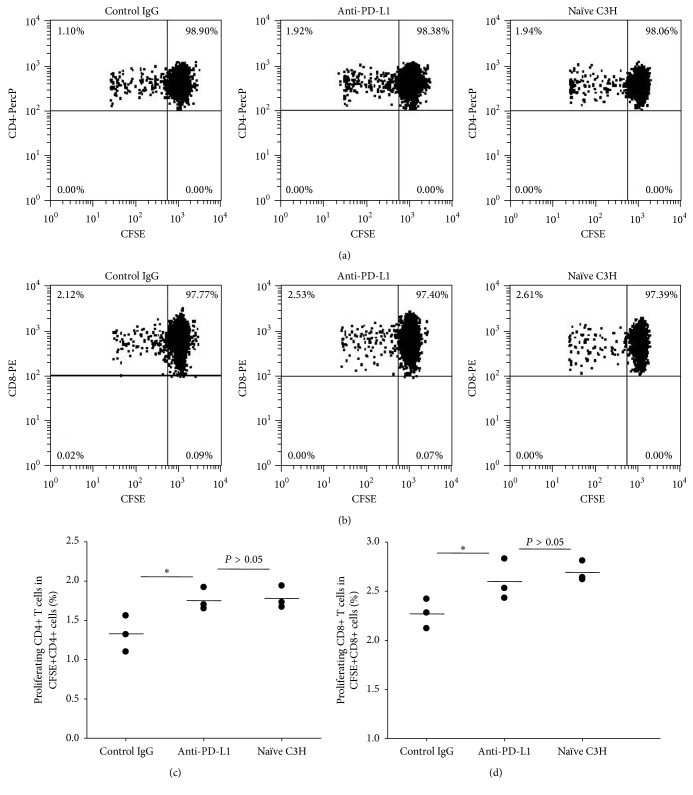
PD-1/PD-L1 interaction controls alloantigen-responding effector T cells in tolerant mice. C3H mice were treated with Balb/c UVB-iDCs to induce alloantigen tolerance as described elsewhere. Six weeks later, CFSE-labeled naïve C3H spleen cells (1 × 10^7^/mouse) together with Balb/c spleen cells (1 × 10^7^/mouse) were intravenously injected into the tolerant mice. One group received intraperitoneal injection of anti-mouse PD-L1 mAb (100 *μ*g/mouse); the other group received control IgG (100 *μ*g/mouse) at the same day when cells were injected. On day 3, the above treatments were administered again. One group of naïve C3H mice was set up only receiving injection of both types of cells. On day 4 after cell injection, all mice were sacrificed and inguinal lymph nodes were collected and prepared for cell suspension. The proliferating CD4+ T cells and CD8+ T cells in CFSE+ lymph node cells were analyzed by flow cytometry. (a) Representative flow cytometric data on CFSE-labeled CD4+ T cell proliferation in each group were shown; (b) representative flow cytometric data on CFSE-labeled CD8+ T cell proliferation in each group were shown; ((c) and (d)) the summary of CD4+ and CD8+ T cell proliferation was shown, respectively. Data represent mean ± standard error. ^*∗*^ *P* < 0.05. The *P* values were calculated using one-way ANOVA.

The results shown above suggest that tolerant T or B cells can be reinvigorated by alloantigen stimulation under PD-1/PD-L1 blockade. It is of interest to learn whether PD-1/PD-L1 interaction is essential in tolerant mice for controlling alloantigen-responding T cells that potentially arise later and never experience alloantigens. Those T cells could lead to allograft rejection or graft-versus-host disease if left unchecked in allogeneic transplantation. To assess whether PD-1/PD-L1 in those alloantigen tolerant mice keeps the alloantigen-responding T cells in check, we adoptively transferred CFSE-labeled naïve C3H spleen cells (syngeneic) along with Balb/c spleen cells (alloantigen) to the tolerant C3H mice or naïve C3H mice. Thereafter, anti-PD-L1 or control IgG was administered to two groups of tolerant mice at day 1 and day 3, respectively. Four days later, we sacrificed the mice and prepared cells from inguinal lymph nodes and assessed the proliferation of injected CFSE-labeled C3H syngeneic CD4+ and CD8+ T cells not preexperiencing alloantigen, in response to injected Balb/c spleen cells (alloantigen)* in vivo*. In comparison to control IgG-treated mice, CD4+ and CD8+ T cell proliferation in response to* in vivo* alloantigen stimulation was significantly enhanced in anti-PD-L1 treated mice, which was similar to the T cell proliferation levels in naïve recipient mice (Figure 6). The above findings indicate that, in tolerant mice, PD-1/PD-L1 interaction plays an important role in keeping potentially arising alloantigen-responding T cells in check and preventing them from being activated.

## 4. Discussion

We previously reported that transfusion of UVB-iDCs was able to induce immune tolerance across MHC barriers and this tolerance was associated with regulatory CD4+ T cells [5, 6]. However, it is unknown whether this tolerance state can be maintained for long term, which is highly desirable for allogeneic transplantation. In the present study, we assessed the tolerance maintenance 6 weeks after finishing tolerance induction. We found that tolerant mice failed to produce any levels of antibodies against alloantigens upon two weekly alloantigen challenges (Figure 1). These results are the same as those observed in the tolerant mice undergoing alloantigen challenge immediately after tolerance induction [6]. These findings suggest that UVB-iDC infusion-induced alloantigen tolerance could be maintained for at least 6 weeks after tolerance induction without any loss. Consistent with these results, we also observed that the tolerant mice failed to reject the intravenously injected CFSE-labeled traceable Balb/c allogeneic spleen cells, which were completely rejected in nontolerant naïve mice (Figure 4) through flow cytometric assessment of peripheral blood. In addition, we found those injected CFSE-labeled allogeneic spleen cells homed to host lymph nodes and spleen in tolerant mice, which did not take place in nontolerant mice (data not shown). This tolerance assessment is a sensitive assay for evaluating allogeneic tolerance* in vivo* by looking at CFSE-labeled cells in the peripheral blood and could be a feasible method for future clinical evaluation of immune tolerance in allogeneic transplantation patients. Allogeneic skin transplantation is usually employed to test allogeneic tolerance. To assess whether tolerant mice 6 weeks after tolerance induction were able to accept allogeneic skin grafts, we performed allogeneic skin transplantation in tolerant and nontolerant mice. It is noted that, without any immunosuppression, the survival of allogeneic skin grafts was significantly prolonged in tolerant mice in contrast to that in nontolerant mice (Figure 3). These data further support that UVB-iDC-induced alloantigen tolerance is quite strong and durable.

We demonstrated previously that, among CD4+ T cells in tolerant mice, there were significantly increased IL-10-producing and Foxp3+ regulatory T cells [5], which may play a crucial role in maintaining tolerance. In the current study, we assessed whether CD4+ T cells in the mice with long-lasting tolerance remained functioning to maintain immune tolerance to alloantigens. We found that the tolerance was transferrable with adoptive CD4+ T cell transfer to syngeneic naïve recipient mice, showing that mice receiving CD4+ T cells from tolerant mice failed to produce antibodies against alloantigens upon alloantigen challenge (Figure 2). Interestingly, the results shown in Figures 1 and 2 are quite similar, indicating that CD4+ T cells exert critical effect on tolerance maintenance. Those CD4+ T cells with regulatory function are likely to be Foxp3+ Tregs and IL-10-producing Tr1 cells [5, 12].

PD-1/PD-L1 interaction has been considered playing an essential role in maintaining peripheral tolerance [7, 8, 13, 14]. Blocking PD-1/PD-L1 interaction can drastically accelerate allograft rejection [15–17]. It was also reported that PD-1/PD-L1 interaction is crucial for maintaining anti-CD3 treatment-induced immune tolerance to islet *β* cell antigens in nonobese diabetic mouse model [18]. Administration of ethylene carbodiimide- (ECDI-) fixed allogeneic splenocytes failed to induce alloantigen tolerance in PD-L1 deficient mice, suggesting that PD-1/PD-L1 is required for alloantigen tolerance induced by ECDI-treated allogeneic splenocytes [11]. In the current study, we were interested in learning whether PD-1/PD-L1 was required for maintaining alloantigen tolerance induced by UVB-iDC treatment. We employed our reliable and reproducible methods and tested the influence of PD-1/PD-L1 blockade by anti-PD-L1 antibodies on immune tolerance to alloantigens upon alloantigen challenge. We showed that PD-1/PD-L1 blockade by anti-PD-L1 antibodies significantly promoted the rejection of infused CFSE-labeled allogeneic spleen cells (Figure 4) compared to control IgG antibody-treated tolerant mice. As expected, the nontolerant naïve mice completely rejected the infused CFSE-labeled allogeneic spleen cells. In line with this finding, the levels of antibodies against alloantigens in response to alloantigen challenge in tolerant mice were largely recovered by the treatment of anti-PD-L1 antibodies but failed to reach the levels of nontolerant naïve mice challenged with alloantigens (Figure 5). The above findings indicate that PD-1/PD-L1 interaction actively participates in maintaining allogeneic tolerance induced by UVB-iDC treatment. Incomplete recovery of immune response to alloantigen challenge might be attributed to incomplete blockade of PD-1/PD-L1 interaction because we only utilized one dose (100 *μ*g/mouse), which might not be the optimal dose to completely block PD-1/PD-L1 interaction. Another possibility is that other tolerance maintenance mechanisms may also be involved. A dose study for anti-PD-L1 antibodies or PD-L1 deficient mouse model may be needed to address whether PD-1/PD-L1 is the only factor to be involved in maintaining the tolerance induced by UVB-iDC treatment.

Finally, we investigated how the tolerance state in tolerant mice affected alloantigen-responding T cells that never experienced alloantigens and whether PD-1/PD-L1 interaction was also involved in this process. We injected CFSE-labeled syngeneic spleen cells from naïve C3H mice into tolerant C3H recipients to serve as responder cells, with simultaneous injection of allogeneic Balb/c spleen cells as allogeneic stimulators. One group was treated with anti-PD-L1 antibodies and the other group received control IgG treatment. As shown in Figure 6, blockade of PD-1/PD-L1 interaction significantly increased the proliferation of injected CFSE-labeled CD4+ and CD8+ T cells, suggesting that PD-1/PD-L1 plays an important role in keeping alloantigen-responding T cells in check. This effect may be through induced alloantigen-specific regulatory T cells as described in our previous study [5]. PD-1 has been reported to be expressed on regulatory T cells [19]. Our data show that CD4+ T cells from UVB-iDC-treated mice express higher levels of PD-1 (Supplemental Figure  1, in Supplementary Material available online at http://dx.doi.org/10.1155/2016/2419621), and those CD4+ T cells might gain regulatory function. Thus, UVB-iDC treatment-induced alloantigen-specific regulatory T cells may control the potential alloantigen-specific effector T cells through PD-1/PD-L1 interaction [20, 21].

Collectively, the present study demonstrates that UVB-iDC infusion-induced allogeneic immune tolerance is long lasting, and PD-1/PD-L1 interaction plays an important role in the maintenance of immune tolerance.

## Supplementary Material

The C3H mice were treated with four weekly injections of UVB-iDCs as described elsewhere. Six weeks later, CD4+ T cells from the treated mice or naïve mice were stained with anti-PD-1 antibodies and the expression of PD-1 on CD4+ T cells was examined by flow cytometry. CD4+ T cells from naïve mice were used as control. A. dot plots show the PD-1+ CD4+ T cells in naïve and treated mice (2 treated mice). B. The histogram in the left shows the expression of PD-1 on CD4+ T cells from a naïve mouse, the histograms in the middle and the right show the histogram overlays of PD-1 expression on CD4+ T cells of UVB-iDC-treated and naïve mice, respectively. The results demonstrate that UVB-iDC treatment induces up-regulation of PD-1 on CD4+ T cells.
