# Supplementary material for: PD-1/PD-L1 Interaction Maintains Allogeneic Immune Tolerance Induced by Administration of Ultraviolet B-Irradiated Immature Dendritic Cells
Source: J Immunol Res. 2016 Jul 31;2016:2419621. doi: 10.1155/2016/2419621 (PMC4983366; doi:10.1155/2016/2419621)
Supplement: Supplementary file 1 — The C3H mice were treated with four weekly injections of UVB-iDCs as described elsewhere. Six weeks later, CD4+ T cells from the treated mice or naïve mice were stained with anti-PD-1 antibodies and the expression of PD-1 on CD4+ T cells was examined by flow cytometry. CD4+ T cells from naïve mice were used as control. A. dot plots show the PD-1+ CD4+ T cells in naïve and treated mice (2 treated mice). B. The histogram in the left shows the expression of PD-1 on CD4+ T cells from a naïve mouse, the histograms in the middle and the right show the histogram overlays of PD-1 expression on CD4+ T cells of UVB-iDC-treated and naïve mice, respectively. The results demonstrate that UVB-iDC treatment induces up-regulation of PD-1 on CD4+ T cells. [file 2419621.f1.pdf]

Supplemental data:

Supplemental Figure 1.

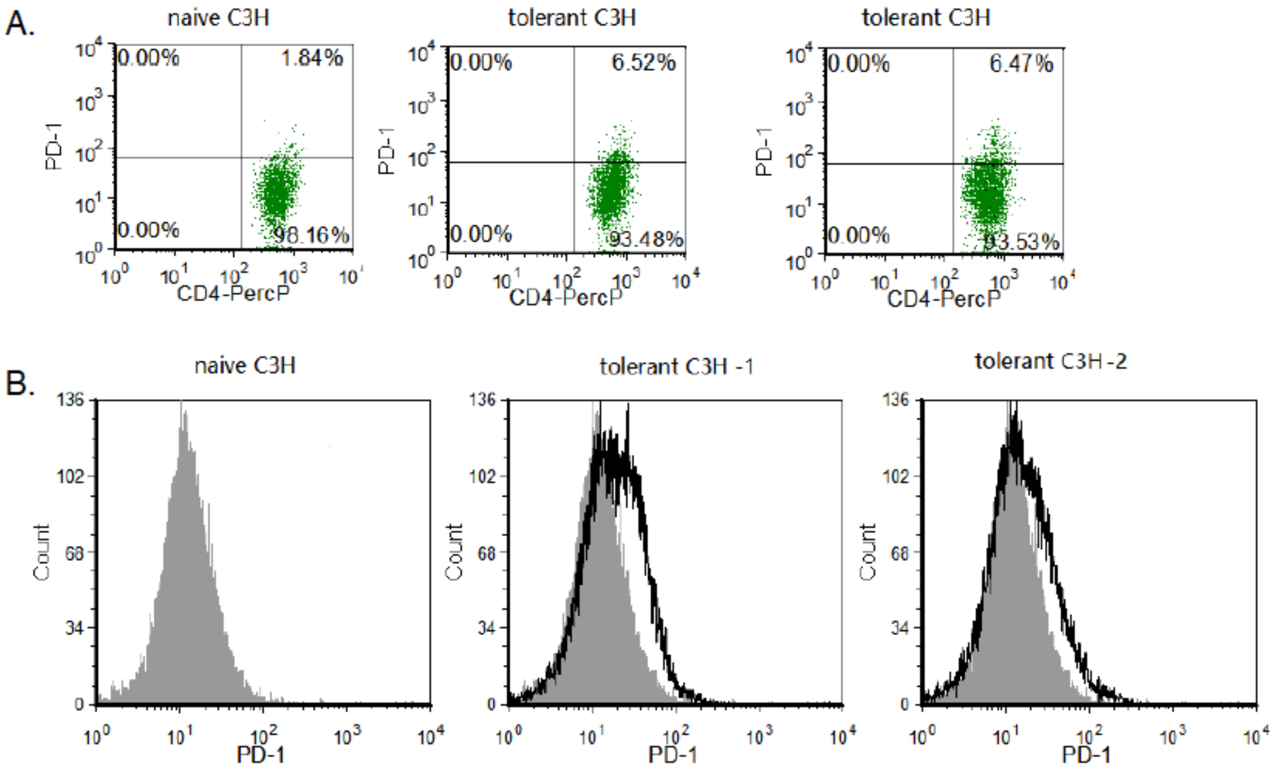

sFig1. CD4+T cells from UVB-iDC treated mice express higher levels of PD-1. The C3H mice were treated with four weekly injections of UVB-iDCs as described elsewhere. Then the CD4+ T cells from the treated mice were stained with anti-PD-1 antibodies and the expression of PD-1 on CD4+ T cells was examined by flow cytometry. CD4+ T cells from naïve mice were used as control. **A.** dot plots show the PD-1+ CD4+ T cells in naïve and treated mice (2 treated mice). **B.** The histogram in the left shows the expression of PD-1 on CD4+ T cells from a naïve mouse, the histograms in the middle and the right show the histogram overlays of PD-1 expression on CD4+ T cells of UVB-iDC-treated and naïve mice, respectively. The results demonstrate that UVB-iDC treatment induces up-regulation of PD-1 on CD4+ T cells.
